# Digital Violence and Abuse: A Scoping Review of Adverse Experiences Within Adolescent Intimate Partner Relationships

**DOI:** 10.1177/15248380231201816

**Published:** 2023-10-11

**Authors:** Stine Torp Løkkeberg, Camilla Ihlebæk, Gudrun Brottveit, Lilliana Del Busso

**Affiliations:** 1 Faculty of Health, Welfare, and Organization, Østfold University College, Fredrikstad, Norway; 2 Faculty of Landscape and Society, Norwegian University of Life Sciences, Ås, Norway

**Keywords:** digital dating abuse, digital dating violence, cyber dating abuse, intimate partner violence, adolescence, a scoping review

## Abstract

International research in the past 2 decades has suggested that intimate partner violence among adolescents is a significant public health concern. Both are commonly understood as a pattern of behavior that is intended to establish and maintain control over a partner. Recently, a plethora of digital applications and social networking sites have presented new opportunities for adolescents to initiate, develop, and conduct intimate partner relationships. However, research exploring adverse experiences related to digital interactions in the context of adolescents’ intimate partner relations is limited. This scoping review aims to identify and describe the nature and range of difficult experiences in the current published research relating to digital interactions between intimate adolescent partners, from digitalized violence to less severe adverse experiences. Systematic and manual searching resulted in the identification of 1,876 potential articles for inclusion in this review. A total of 18 articles were ultimately included based on the following predefined inclusion criteria. The article must: (a) be an empirical study that has used quantitative, qualitative, mixed, or review methods; (b) include young adolescents and adolescents of 18 years or younger as participants; (c) include accounts of young adolescents and young people’s experiences and/or consequences of digital interactions within intimate partner relationships; and (d) be published in a peer-reviewed journal. Examples of less severe experiences could be different kinds of digital harassment, such as electronic intrusiveness, excessive texting, insults, unpleasant messages, and the spreading of rumors. Other adverse experiences related to digital interactions included being controlled by a partner, verbal abuse, experiences of aggression, sexual pressure, and coercion. Common consequences of adverse experiences included emotional and mental health-related difficulties, self-restricting behaviors, relationship difficulties, and risk behaviors.

## Introduction

The exploration of intimate, romantic, and sexual interactions and relationships with peers usually starts in early adolescence, which is a critical time for physical, emotional, and psychosocial development (Caridade & Dinis, 2020; Leadbeater et al., 2018). Current international research, however, indicates that a considerable number of adolescents endure physically and psychologically harmful experiences within their intimate partner relationships (Aghtaie et al., 2017; Park et. al., 2018; Stonard et al., 2014). The World Health Organization (WHO) has defined intimate partner violence (IPV) as “behaviour by an intimate partner or ex-partner that causes physical, sexual or psychological harm, including physical aggression, sexual coercion, psychological abuse and controlling behaviours” (WHO, 2021). In doing so, WHO (2021) draws on prevalence research from 161 countries and emphasizes that women and girls are disproportionally affected by IPV. Nevertheless, regardless of their sexuality and gender identity, adolescents may experience intimate relationships characterized by precise or subtle forms of physical and psychological abuse. The harmful actions of an intimate partner may thus be complex and varied and simultaneously include physical, emotional, and psychological aspects (Leadbeater et al., 2018).

Furthermore, the emergence of digital tools and platforms presents new opportunities for adolescents to initiate, develop, and conduct relationships (Borrajo, 2020). This may contribute to the complexity of IPV and its consequences. Digitalization may compound the problem of IPV—for example, through the increase in points and forms of contact between partners (e.g., sexting, “Facetiming,” and sending intimate photos and videos on a smartphone), and the opportunity to gain insight into a partner’s private life, interactions with others, and activities through social media (Henry & Powell, 2015 **).** In the context of traditional understandings of IPV, Vale et al. (2020, p. 89) suggest that digital dating violence (DDV) “should be understood as an innovative, versatile, ubiquitous, extensive and efficient strategy, compared to adolescents’ conventional dating abuse.” It can encompass many forms of abuse, from online harassment, hate speech, doxing, cyber stalking, and image-based abuse, to gendered disinformation.

Digital dating abuse (DDA) often constitutes harmful behaviors that are carried out by one partner toward the other remotely—not targeting the physical body of the other partner directly, but by utilizing technology (such as a smartphone, social media, a tracking app or another form of technology) to “blackmail, control, coerce, harass, humiliate, objectify or violate” (Henry et al., 2015, p. 398) the other person.

Such “remote” and seemingly disembodied digitalized behaviors can have severe negative, physical, and emotional consequences. Experiencing that a current or previous intimate partner has taken and/or shared a digital sexual image of oneself without consent, for example, can have a negative impact on short- and long-term health, and may also contribute to a decline in mental and physical health, including self-harm and suicide (Buiten, 2020; Henry & Powell, 2015; Naezer & van Oosterhout, 2021). WHO (2021) suggests that adolescence is essential for laying the “foundations for good health.” Experiencing DDA during this period, which is characterized by significant physical, emotional and psychological developmental changes, means that adolescents may be less resourceful than most adults in dealing with such adverse experiences (Cutter-Wilson et al., 2011; Draucker et al., 2012).

The prevalence of DDV and DDA among adolescents is difficult to estimate. For example, a review by Caridade et al. (2020) found that the reported prevalence of cyber dating abuse victimization varied between 5.8% and 92% in different studies. Many studies have investigated the risk factors for DDV and DDA (Caridade et al., 2020; Gassó et al., 2019), but less is known about the consequences of such adverse experiences. In line with the international recognition of adolescent IPV as a considerable public health concern and the further complexity presented by the rapid digitalization of communication and interaction, our aim in this scoping review was to identify and describe the nature and range of adverse experiences and consequences explicitly related to digital interactions between intimate adolescent partners in the current published research.

## Method

### Search Strategies

A senior university librarian conducted systematic searches in ProQuest, CINAHL, PubMed, Embase, and PsychInfo. Search terms were used to describe the phenomenon of interest (e.g., IPV, dating violence, sexual abuse, adverse experience), population (e.g., child, adolescent, teenager), type of relationship (e.g., intimate partner interaction, dating, boyfriend–girlfriend dyad, friends with benefits, hooking up, flirting), and mode of digital interaction (e.g., cyber dating, technology-facilitated dating, sexting). In addition, the four authors of this review conducted manual searches in relevant journals, reference lists of articles that were considered to be of key importance, and appropriate author publication lists.

### Inclusion and Exclusion Criteria

To identify articles that were eligible for this review, the following inclusion criteria were utilized. The article must: (a) be an empirical study that has used quantitative, qualitative, mixed, or review methods; (b) include young adolescents and adolescents of 18 years or younger as participants; (c) include accounts of young adolescents and young people’s experiences and/or consequences of digital interactions within intimate partner relationships; and (d) be published in a peer-reviewed journal. Our criteria for excluding studies from the review were: (a) studies published in a non-English language; (b) books, book chapters, conference proceedings, and gray literature; (c) studies that explore adult sexual abuse of young adolescents and adolescents; (d) intervention studies; (e) validation studies (tools, measures); and (f) studies where experiences were used to predict DDV or DDA or where the causal relationships were unclear.

### Source of Evidence Screening and Selection

**Figure 1.**
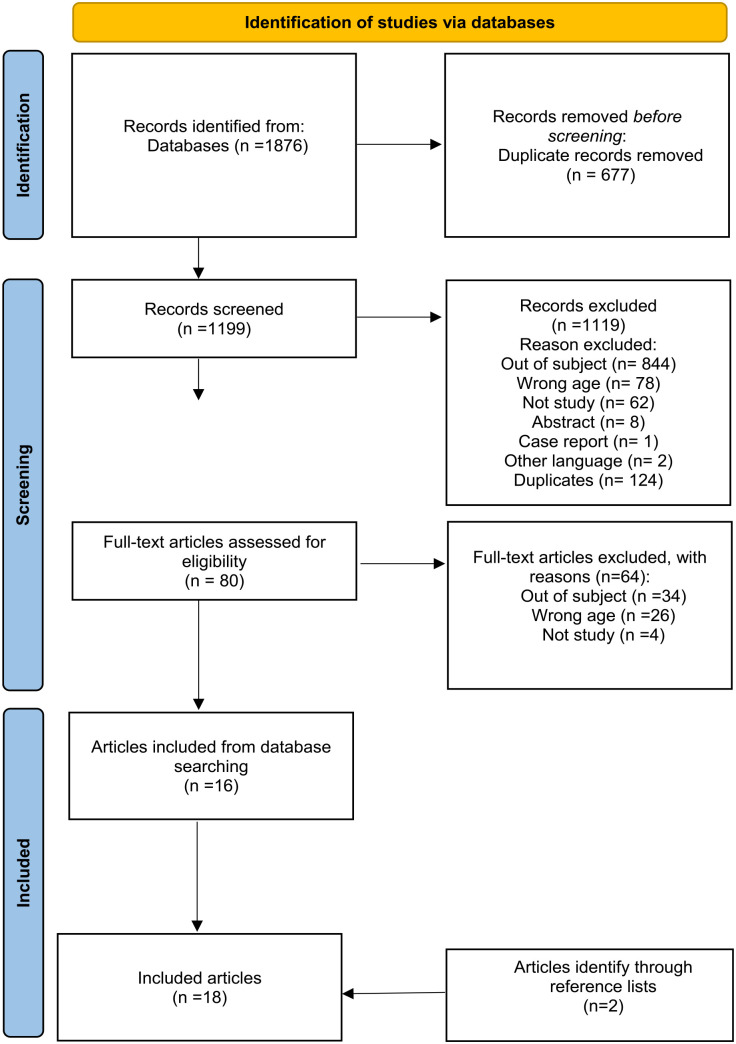
Flow diagram of the selection process.

Systematic and manual searching resulted in 1,876 potentially relevant articles. The articles were imported into Rayyan QCRI, which is a systematic reviews web application (Ouzzani et al., 2016). After removing duplicates, 1,199 articles were eligible for review (Figure 1). In the first screening stage, article titles, abstracts, and keywords were independently assessed by four team members, divided into two teams. Each team was allocated half of the identified articles. Then, within each team, two authors assessed the articles in Rayyan in blinded mode, compared their results, and agreed on whether or not an article should be included or excluded.

This process resulted in the identification of 80 potentially relevant articles. In the next screening stage, the texts of these 80 articles were read in full (Figure 1). Each of the two teams read 40 articles. Within each team, two authors independently read and assessed each article according to the inclusion and exclusion criteria, before finally comparing their results. Any disagreements and uncertainties were resolved through discussions among all four authors. This process resulted in the exclusion of 64 articles, based on the following categories: Out of subject (*n* = 34); Wrong age (*n* = 26); and Not study (*n* = 4), which left 16 articles to be included in the review.

In the final screening stage, we distributed the remaining 16 included articles among ourselves, and screened the articles’ reference lists. Based on this process, two new articles were included. After the last screening, we were left with 18 included articles (see Figure 1).

Table 1 gives an overview of the included articles.

**Table 1.** Overview of the Included Studies.

Study (Ref. No.)	Study Population and Age	Methods	Sample Size	Geographical Area
Baker (2017), What role do peers play in adolescent dating? Insights from adolescents with a history of dating violence	Teenagers, 14–18 years	Qualitative, focus group	(*n* = 39)	USA/Hawaii
Baker and Carreño (2016), Understanding the role of technology in adolescent dating and dating violence	Youth, 14–18 years	Qualitative, focus group	(*n* = 39)	USA/Hawaii
Barter et al. (2017), Young people’s online and face-to-face experiences of interpersonal violence and abuse and their subjective impact across five European countries	Young people, 14–17 years	Quantitative, survey	(*n* = 4.564)	Europe
Cava et al. (2020), Loneliness, depressive mood and cyberbullying	Adolescents, 12–17 years	Quantitative, survey	(*n* = 1,063)	Spain
Doucette et al. (2021), Perpetration of electronic intrusiveness among adolescent females: associations with in-person dating violence	Adolescents, 14–17 years	Quantitative, survey	(*n* = 109)	USA
Goebert et al. (2011), The impact of cyberbullying on substance use and mental health in a multi-ethnic sample	High school students, 14–18 years	Mixed methods, focus group and survey	(*n* = 677)	USA/Hawaii
Hellevik (2019), Teenagers’ personal accounts of experiences with digital intimate partner violence and abuse	Teenagers, 15–18 years	Qualitative, in-depth interviews	(*n* = 14)	Norway
Hinduja et al. (2020), Digital dating abuse among a national sample of U.S. youth	Middle and high school students, 12–17 years	Quantitative, survey	(*n* = 2,218)	USA
Lucero et al. (2014), Exploring gender differences: socially interactive technology use/abuse among dating teens	Highschool teens, 12–18 years	Qualitative, focus groups	(*n* = 23)	USA
Ortega-Baron et al. (2022), Epidemiology of cyber dating abuse victimization in adolescence and its relationship with health-related quality of life: a longitudinal study	Adolescents, 14–18 years	Quantitative, questionnaire	(W1: *N* = 341; W2: *N* = 357; W3: *N* = 416)	Spain
Reed et al. (2017), Gender matters: experiences and consequences of digital dating abuse victimization in adolescent dating relationships	Adolescents, 13–19 years	Quantitative, survey	(*n* = 703)	USA
Reed et al. (2020), Name-calling, jealousy, and break-ups: teen girls’ and boys’ worst experiences of digital dating	Boys and girls, 14–18 years	Quantitative, survey	(*n* = 262)	USA
Rueda et al. (2015), “She posted it on facebook”: Mexican American adolescents’ experiences with technology and romantic relationship conflict	Adolescent, 15–17 years	Mixed qualitative methods	(*n* = 64, *n* = 34, *n* = 14)	USA
Smith et al. (2018), Cyber dating violence: prevalence and correlates among high school students from small urban areas in Quebec	Boys and girls, 14–17 years	Quantitative, survey	(*n* = 398)	Canada
Stanley et al. (2018), Pornography, sexual coercion and abuse and sexting in young people’s intimate relationships: a European study	Young students, 14–17 years	Mixed quantitative and qualitative methods	(*n* = 4,564)	Bulgaria, Cyprus, England, Italy, and Norway
Stonard et al. (2017), They’ll always find a way to get to you: technology use in adolescent romantic relationships and its role in dating violence and abuse	Secondary school, 12–18 years	Qualitative methods, focus groups	(*n* = 52)	UK
Ouytsel et al. (2019), Adolescents’ perceptions of digital media’s potential to elicit jealousy, conflict and monitoring behaviors within romantic relationships	Secondary school students, 15–18 years	Qualitative, focus group	(*n* = 55)	Belgium
Zweig et al. (2013), The rate of cyber dating abuse among teens and how it relates to other forms of teen dating violence	Youth, 13–18 years	Quantitative survey	(*n* = 3,745)	USA

*Note.* Table summary of the included articles.

In line with Peters et al. (2015), we did not conduct a quality appraisal as, in contrast to a systematic review, scoping reviews are “designed to provide an overview of the existing evidence base regardless of quality” (p. 142).

## Results

### General Characteristics of the Included Empirical Studies

This review showed that research concerning adverse digital experiences within adolescent intimate partner relationships has mainly been conducted using quantitative methods (Table 2). The most common design was a cross-sectional survey; only one study had a longitudinal design. A qualitative design was used in six studies, and four studies involved the application of mixed methods. In the majority of the studies, the study populations included both males and females. One study focused on females only, and three studies included gender expressions other than male and female (Table 2). In more than half of the empirical studies, sexuality was not specified. In seven studies, all sexualities or sexual relationships were included, and in one study, only heterosexual relationships were included. Most studies included study populations from a high school, equivalent to the age group 14 to 18 years. However, some studies also included younger adolescents, down to the age of 13 years. All included studies were conducted in North America or Europe (Table 2).

**Table 2.** Characteristics of the Included Articles.

Characteristics	*n*	Articles
Study design		
Quantitative	8	
Cross-sectional	7	Barter et al. (2017), Cava et al. (2020), Doucette et al. (2021), Hinduja and Patchin (2020), Reed et al. (2017), Smith et al. (2018), and Zweig et al. (2013)
Longitudinal	1	Ortega-Barón et al. (2022)
Qualitative	6	
Individual interviews	1	Hellevik (2019)
Focus groups	5	Baker (2017), Baker and Carreño (2016), Lucero et al. (2014), Stonard et al. (2017), and Van Ouytsel et al. (2019)
Mixed methods	4	Goebert et al. (2011), Reed et al. (2020), Rueda et al. (2015), and Stanley et al. (2018)
Study population		
Gender		
Male and female	14	Baker (2017), Baker and Carreño (2016), Barter et al. (2017), Cava et al. (2020), Goebert et al. (2011), Hellevik (2019), Hinduja and Patchin (2020), Lucero et al. (2014), Ortega-Barón et al. (2022), Rueda et al. (2015), Smith et al. (2018), Stanley et al. (2018), Stonard et al. (2017), and Van Ouytsel et al. (2019)
Female	1	Doucette et al. (2021)
All genders	3	Reed et al. (2017), Reed et al. (2020), and Zweig et al. (2013)
Sexuality		
Heterosexual relationships	1	Rueda et al. (2015)
All sexual relationships	7	Barter et al. (2017), Cava et al. (2020), Doucette et al. (2021), Hinduja and Patchin (2020), Reed et al. (2017), Reed et al. (2020), and Zweig et al. (2013)
Not specified	10	Baker (2017), Baker and Carreño (2016), Goebert et al. (2011), Hellevik (2019), Lucero et al. (2014), Ortega-Barón et al. (2022), Smith et al. (2018), Stanley et al. (2018), Stonard et al. (2017), and Van Ouytsel et al. (2019)
Age		
12–17	2	Cava et al. (2020) and Hinduja and Patchin (2020)
12–18	1	Stonard et al. (2017)
13–18	1	Ortega-Barón et al. (2022)
13–19	1	Reed et al. (2017)
14–17	3	Barter et al. (2017), Doucette et al. (2021), and Stanley et al. (2018)
14–18	6	Baker (2017), Baker and Carreño (2016), Goebert et al. (2011), Reed et al. (2020), Smith et al. (2018), and Zweig et al. (2013)
15–17	2	Lucero et al. (2014) and Rueda et al. (2015)
15–18	2	Hellevik (2019) and Van Ouytsel et al. (2019)
Geographical distribution		
North America	11	Baker (2017), Baker and Carreño (2016), Doucette et al. (2021), Goebert et al. (2011), Hinduja and Patchin (2020), Lucero et al. (2014), Reed et al. (2017), Reed et al. (2020), Rueda et al. (2015), Smith et al. (2018), and Zweig et al. (2013)
Europe	7	Barter et al. (2017), Cava et al. (2020), Hellevik (2019), Ortega-Barón et al. (2022), Stanley et al. (2018), Stonard et al. (2017), and Van Ouytsel et al. (2019)

### Adolescents’ Adverse Experiences Within Intimate Partner Relationships

The included empirical studies reported various adverse experiences associated with digital intimate partner relationships. The adverse experiences ranged from less severe, such as sending or posting insults or unpleasant messages, to more severe experiences, such as blackmailing, emotional violence, and sharing nude photos. Four main themes of adverse experiences were identified (Table 3). First, *aggression* included different severe forms of adverse experiences where a partner subjected another to aggressive behavior via digital platforms, such as threatening messages (e.g., Doucette et al., 2021; Goebert et al., 2011; Hinduja & Patchin, 2020; Reed et al., 2017; Zweig et al., 2013), blackmailing behavior (Hellevik, 2019; Smith et al., 2018), cyber bullying (Cava et al., 2020), or other forms of aggressive or violent behavior (e.g., Barter et al., 2017; Ortega-Barón et al., 2022; Reed et al., 2020; Table 3). One of the most commonly reported adverse digital experiences within digital intimate relationships was being subjected to *controlling behavior* by a partner (e.g., Baker, 2017; Baker & Carreño, 2016; Cava et al., 2020; Hinduja & Patchin, 2020; Ortega-Barón et al., 2022; Stanley et al., 2018; Stonard et al., 2017; Van Ouytsel et al., 2019). The controlling behavior could consist of monitoring the partner’s phone or social media (e.g., Doucette et al., 2021; Hellevik, 2019; Lucero et al., 2014; Rueda et al., 2015; Reed et al., 2017, 2020) or surveillance of their social media activity (Barter et al., 2017; Rueda et al., 2015; Table 3).

**Table 3.** Adverse Digital Experiences. Themes and Theme Descriptions Identified in the Included Articles.

Theme	Theme Description	Articles
Aggression	Threats, blackmail, bullying, aggression, and emotional violence	Barter et al. (2017), Cava et al. (2020), Doucette et al. (2021), Goebert et al. (2011), Hellevik (2019), Hinduja and Patchin (2020), Ortega-Barón et al. (2022), Reed et al. (2017), Reed et al. (2020), and Smith et al. (2018), Zweig et al. (2013)
Controlling behavior	Controlling behavior, monitoring, and surveillance	Baker (2017), Baker and Carreño (2016), Barter et al. (2017), Cava et al. (2020), Doucette et al. (2021), Goebert et al. (2011), Hellevik (2019), Hinduja and Patchin (2020), Lucero et al. (2014), Ortega-Barón et al. (2022), Reed et al. (2017), Reed et al. (2020), Rueda et al. (2015), Stanley et al. (2018), and Stonard et al. (2017), Van Oytsel et al. (2019)
Harassment	Electronic intrusiveness, violations of privacy, excessive texting, insults, unpleasant messages/posts, spreading rumors, outing, and scary messages	Baker (2017), Baker and Carreño (2016), Barter et al. (2017), Cava et al. (2020), Doucette et al. (2021), Goebert et al. (2011), Hellevik (2019), Hinduja and Patchin (2020), Lucero et al. (2014), Ortega-Barón et al. (2022), Reed et al. (2017), Reed et al. (2020), Rueda et al. (2015), Smith et al. (2018), Stonard et al. (2017), and Zweig et al. (2013)
Pressure and coercion	Sexual pressure, sexual threats, sexual coercion, sharing private texts, pictures, and nude photos	Hellevik (2019), Hinduja and Patchin (2020), Ortega-Barón et al. (2022), Reed et al. (2017), Reed et al. (2020), Stanley et al. (2018), Smith et al. (2018), and Zweig et al. (2013)

Different forms of digital *harassment* were also reported and covered a wide range of adverse experiences (Table 3). These experiences might not be regarded as being as severe as the experiences of aggression and control, but they were commonly reported and included electronic intrusiveness (e.g., Baker, 2017; Baker & Carreño, 2016; Cava et al., 2020; Doucette et al., 2021), violations of privacy (Lucero et al., 2014; Zweig et al., 2013), excessive texting (e.g., Hellevik, 2019; Reed et al., 2020; Rueda et al., 2015; Stonard et al., 2017), insults (e.g., Ortega-Barón et al., 2022; Smith et al., 2018), unpleasant messages or posts (e.g., Barter et al., 2017; Hinduja & Patchin, 2020), being the subject of rumors (e.g., Goebert et al., 2011; Reed et al., 2017; Smith et al., 2018), outing of private information (Barter et al., 2017; Ortega-Barón et al., 2022; Smith et al., 2018), and sending frightening messages (Hellevik, 2019; Table 3).

Some studies found that adolescents had experienced *pressure and coercion* through digital platforms from their intimate partner (Table 3). This was often related to sexual pressure (Hellevik, 2019; Reed et al., 2017, 2020; Zweig et al., 2013), sexual threats (Hellevik, 2019; Zweig et al., 2013), or sexual coercion (Hellevik, 2019; Reed et al., 2017, 2020; Stanley et al., 2018). Several studies also reported that adolescents in intimate digital relationships had experienced pressure to share private texts, pictures, and nude photos (Hellevik, 2019; Hinduja & Patchin, 2020; Ortega-Barón et al., 2022; Reed et al., 2017, 2020; Smith et al., 2018; Zweig et al., 2013; Table 3).

### Consequences of Adverse Experiences

The results of this review suggest that having adverse digital experiences within intimate partner relationships as an adolescent can have several consequences, ranging from an effect on relationship dynamics to risk behaviors, self-harm, and health consequences (Table 4). In addition, the digital communication and social media platforms were reported to be factors that facilitated different forms of *constraints on the intimate relationship*, such as break-ups (Reed et al., 2017, 2020), conflicts (e.g., Lucero et al., 2014; Van Ouytsel et al., 2019), irritation (Baker 2017; Stonard et al., 2017; Van Ouytsel et al., 2019), jealousy (e.g., Baker, 2017; Baker & Carreño, 2016; Rueda et al., 2015), distrust (Lucero et al., 2014; Rueda et al., 2015), and misunderstandings (Rueda et al., 2015; Table 4).

**Table 4.** Consequences of Adverse Digital Experiences. Themes and Theme Descriptions Identified in the Included Articles.

Theme	Theme Description	Articles
Relationship consequences	Break-ups, conflicts, irritation, jealousy, distrust, and misunderstandings	Baker (2017), Baker and Carreño (2016), Lucero et al. (2014), Reed et al. (2017), Reed et al. (2020), Rueda et al. (2015), Stonard et al. (2017), and Van Oytsel et al. (2019)
Self-restricting behavior	Avoidance, isolation, restricting self-expression, or social contact	Baker (2017), Baker and Carreño (2016), Barter et al. (2017), Hellevik (2019), Reed et al. (2017), Reed et al. (2020), and Van Oytsel et al. (2019)
Self-harm	Self-harm, revictimization, and suicide attempts	Goebert et al. (2011) and Hellevik (2019)
Risk behaviors	Substance abuse	Goebert et al. (2011)
Emotional and health-related difficulties	Embarrassment, humiliation, insecurity, low self-esteem, loneliness, depressive mood, emotional or psychological distress, fear and anxiety, low emotional well-being, reduced quality of life, and sleeping problems	Baker (2017), Baker and Carreño (2016), Cava et al. (2020), Doucette et al. (2021), Goebert et al. (2011), Hellevik (2019), Ortega-Barón et al. (2022), Reed et al. (2017), Reed et al. (2020), Smith et al. (2018), Stanley et al. (2018), and Stonard et al. (2017)

Additional consequences of adverse digital experiences, especially in the form of a partner’s aggression or controlling behavior, were different forms of *self-restricting behavior*, such as avoidance of the partner or social media engagement (Reed et al., 2017, 2020; Van Ouytsel et al., 2019), isolation (Baker, 2017; Baker & Carreño, 2016; Barter et al., 2017), or restrictions of self-expression or social contact with others (Hellevik, 2019; Table 4).

Two studies reported that *self-harm* and *risk behaviors* could be a consequence of digital intimate partner abuse (Goebert et al., 2011; Hellevik, 2019). Hellevik (2019) reported that digital IPV could lead to revictimization, caused by the repeated rereading of abusive messages. Additional serious consequences such as suicide attempts (Goebert et al., 2011; Hellevik, 2019) and substance abuse (Goebert et al., 2011) were also reported in the literature (Table 4).

Several studies reported *emotional and health-related consequences* of adverse digital experiences within intimate partner relationships. For example, many adolescents had experienced feelings of embarrassment (Reed et al., 2017, 2020), humiliation (Hellevik, 2019; Stanley et al., 2018), insecurity (e.g., Baker, 2017; Baker & Carreño, 2016), reduced self-esteem (Baker, 2017; Smith et al., 2018), or loneliness (Cava et al., 2020) following adverse experiences. Some studies also reported more severe emotional and psychological problems, such as depressive mood (e.g., Cava et al., 2020; Doucette et al., 2021; Goebert et al., 2011), emotional and psychological distress (Reed et al., 2017, 2020; Smith et al., 2018; Stonard et al., 2017), fear and anxiety (Stonard et al., 2017), low emotional well-being (Stonard et al., 2017), and reduced quality of life (Ortega-Barón et al., 2022). One study also reported sleeping difficulties as a consequence of adverse digital experiences (Hellevik, 2019).

## Discussion

The majority of the 18 studies included in this review used a quantitative cross-sectional approach, and all were conducted in North America or Europe. The results showed that adolescents experience a variety of adverse experiences through digital interactions within their intimate partner relationships, and that such experiences may have considerable consequences for their physical, emotional, and mental health.

### Adverse Experiences

Overall, the most common adverse experience among adolescents identified in the studies included in this review was being controlled by a partner (Baker, 2017; Baker & Carreño, 2016; Barter et al., 2017; Cava et al., 2020; Goebert et al., 2011; Hellevik, 2019; Hinduja & Patchin, 2020; Reed et al., 2017; Rueda et al., 2015; Stanley et al., 2018; Van Ouytsel et al., 2019). This included being monitored and surveilled. In a study by Hellevik (2019), for example, several participants stated that their partner had pressured them to block or delete friends or acquaintances. Further, taking control over the person’s life was related to holding of passwords and deleting pictures, posts, and social media accounts.

Furthermore, harassing experiences such as electronic intrusiveness, violations of privacy, receiving an excessive number of texts from a partner, or receiving messages that included insults or content experienced as frightening were common (Baker & Carreño, 2016; Barter et al., 2017; Doucette et al., 2021; Hellevik, 2019; Ortega-Barón et al., 2022; Reed et al., 2017, 2020; Rueda et al., 2015; Smith et al., 2018). Other difficult experiences included forms of aggression such as threats, blackmail, bullying, and psychological violence (Barter et al., 2017; Cava et al., 2020; Hinduja & Patchin, 2020; Ortega-Barón et al., 2022; Reed et al., 2017, 2020; Smith et al., 2018; Zweig et al., 2013). Finally, many adolescents reported having been coerced into sexual activity, pressured to share pictures of themselves that included nudity, and having had their nude images shared without consent (Hellevik, 2019; Hinduja & Patchin, 2020; Reed et al., 2017, 2020; Zweig et al., 2013).

Although the acts of, for example, control, aggression, and coercion represented in the research included in this review were carried out and experienced through digital modes of interaction, they cannot necessarily be considered disembodied or less harmful than traditional forms of aggression and abuse. Indeed, these incidents should be regarded as embodied experiences that can be felt and sensed emotionally, psychologically and bodily (Buiten, 2020; Henry & Powell, 2015; Naezer & van Oosterhout, 2021). In line with these ideas, Kirkengen and Næss (2021) have shown that young people who have experienced nonphysical, emotional, and psychological abuse have a greater risk of long-term health challenges and ill-health in adulthood (Kirkengen & Næss, 2021). Furthermore, the research included and the adverse experiences identified in this review are in line with the assertion made by Vale et al. (2020) that, compared to traditional, non-digital modes of abuse, DDV is a “versatile, ubiquitous, extensive and efficient strategy” for causing harm to another person. Nevertheless, a limitation of the currently existing knowledge concerning adolescents’ *experiences* of DDA/DDV is the lack of empirical research that has utilized qualitative research methodologies. There is a lack of research exploring adolescents’ accounts of what it is like to live through the experiences of DDA/DDV. To further develop current understandings of adolescents’ experiences of DDA/DDV and to design effective preventative measures and interventions, it can therefore be argued that it would be beneficial to explore how adolescents themselves make sense of and understand their experiences through first-person accounts that are rich in detail in the context of qualitative research.

### Consequences of Experiencing Digital Violence

The most commonly reported consequence of experiencing DDA in the studies included in this review were difficulties relating to physical, emotional, and mental health (Baker, 2017; Baker & Carreño, 2016; Cava et al., 2020; Doucette et al., 2021; Goebert et al., 2011; Hellevik, 2019; Hinduja & Patchin, 2020; Ortega-Barón et al., 2022; Reed et al., 2017, 2020; Smith et al., 2018; Stanley et al., 2018). Such consequences included reduced self-esteem, insecurity, depressive moods, emotional distress, fear and anxiety, reduced quality of life, and sleeping problems. In addition, two studies reported self-harm and attempted suicide as consequences of experiencing DDA (Goebert et al., 2011; Hellevik, 2019).

Another consequence reported in several of the included studies was self-restricting behavior, such as a sense of loss of autonomy, restricting one’s self-expression, or self-isolating and withdrawing from social contact with others (Baker, 2017; Baker & Carreño, 2016; Barter et al., 2017; Hellevik, 2019; Reed et al., 2020). Finally, several studies included in this scoping review reported relationship difficulties due to DDA/DDV (Baker, 2017; Baker & Carreño, 2016; Reed et al., 2020; Rueda et al., 2015; Van Ouytsel et al., 2019). Examples of this included break-ups, conflicts, jealousy, misunderstandings within intimate relationships, and the loss of other relationships, such as important friendships. In line with relationship difficulties being cited as a consequence of experiencing DDA/DDV in the literature reviewed here, it has been suggested that adolescents who have experienced violence in a relationship have an increased risk of perpetrating violence in relationships and engaging in intimate relationships in which mutual violence takes place (Cutter-Wilson & Richmond, 2011; Øverlien, 2020; Park et al., 2018). Overall, the review presented in this article indicates that experiencing DDA/DDV as a child or adolescent can have severe health-related short- and long-term consequences.

However, a significant limitation identified in this scoping review is the lack of longitudinal studies that assess the long-term effects of experiencing specific forms of DDA/DDV in childhood or adolescence.

### Gender Differences

Although our aim was not to dichotomize gender, there are some interesting gender differences we need to address. For example, Baker and Carreño (2016) did not find any differences in the actual use of technology; rather the distinction was related to reasons for their behavior. For example, girls used technology to get to know a boy before in-person contact occurred. For boys, it was a face-saving mechanism in case their hook-up attempt was unsuccessful. They monitored girls to ensure the potential girlfriend was not meeting other boys (Baker & Carreño, 2016). Girls were also more likely than boys to attribute negative impacts to their experiences and reported that they felt scared or upset (Barter et al., 2017). These latter findings are consistent with those of Lu et al., (2020) who found that boys report more victimization with regard to in-person dating violence than DDV. Conversely, the boys in Barter et al. (2017) stated an emotional impact or no effect, and their most negative response was to feel annoyed (Barter et al., 2017). This is in line with González-Cabrera et al. (2021), who also found higher frequency in boys who had not suffered any risk, and girls reported higher prevalence of low health-related quality of life compared to boys. Interestingly, Froidevaux et al. (2022) found no gender differences in average rates of either dating violence perpetration or victimization.

### Limitations

This scoping review has strengths and limitations that should be considered when interpreting the results. First, the search was limited to literature published in English, and only included peer-reviewed studies. Thus, potentially relevant gray literature might have been included. Second, there was no appraisal of the methodological quality of the included studies (Peters et al., 2015), and studies with varying degrees of quality may therefore be included in this scoping review. Nevertheless, the criterion of only including research published in peer-reviewed journals should ensure an acceptable quality regarding the articles included. Third, as most of the articles had a cross-sectional design, we had to identify how different authors had presented and interpreted the causal relationships in their studies, and whether experiences or consequences were presented as the outcome rather than the predicting factors. To ensure high quality throughout this review’s search, screening, and summarization processes, all methodological steps were carried out by a team of researchers who independently reviewed and assessed the records.

A further limitation of the research identified in this scoping review is that half of the studies included did not specify the sexual orientation of the research participants; in the other half, all sexualities or sexual relationships were specified, except for one study, which only included heterosexual relationships. Regarding DDA/DDV among adolescents, a lack of attention to sexual orientation and identity is highly problematic (Øverlien et al., 2020). Not paying attention to the specifics of sexual orientation and identity in a study (e.g., only including heterosexual participants without acknowledging this aspect of the research in the interpretation of the results) can serve to generalize characteristics of DDA/DDV that are not easily generalizable to adolescents who do not identify as heterosexual. Within this field of research, therefore, the diversity of lived experiences (e.g., about sexual and embodied practices and available digital tools and platforms) should be acknowledged and emphasized to provide nuanced knowledge that can be utilized to understand and prevent DDA/DDV, as well as to provide suitable interventions for adolescents tailored to their specific circumstances and needs.

### Implications

The findings of this scoping review highlight the need for research that provides nuanced and in-depth knowledge about digitalized violence within adolescent intimate partner relationships to prevent abuse, and provide young people with guidance regarding interacting with others digitally. As demonstrated, a significantly higher number of studies have employed a quantitative design than a qualitative one. For sensitive research topics such as experiences of abuse, it may be easier to collect data via extensive surveys, where the respondents are less “visible” to the researcher (Øverlien et al., 2020). In most cases, filling out a questionnaire will certainly be less taxing and burdensome than having to meet with a researcher and talk about personal experiences of living through abuse.

According to Østby and Stefansen (2017), there are many mechanisms (e.g., stigma, exclusion, shame) that influence whether young people will tell someone about their experiences. The cross-sectional design of most studies within this field of research also makes it difficult to conclude causal relationships. Therefore, to fully understand how adverse digital experiences within intimate partner relationships affect adolescents, and the negative consequences such experiences might have, there is a need for longitudinal studies and studies that utilize qualitative research methods (Tables 5 and 6).

**Table 5.** Summary of Critical Findings in the Scoping Review: Adverse Experiences and Consequences. Key Findings.

• Many adolescents experience a range of mild to severe adverse digital interactions within intimate partner relations • Negative digital experiences can have negative consequences for adolescents’ physical, emotional, and mental health • As a field of research this area could benefit from studies that employ longitudinal and qualitative research methods to produce nuanced and in-depth knowledge about the adverse digital experiences of adolescents within intimate partner relations, and the long-term consequences of such adverse experiences on child and adolescent health • Adverse experiences in the context of digital interactions included being controlled by a partner, experiences of aggression and violence, digital harassment, and pressure and coercion

*Note.* Summary of the critical findings in the scoping review.

**Table 6.** Summary of critical findings in the scoping review: Practice, policy, and research. Implications.

• Although the acts of, for example, control, aggression, and coercion represented in the research included in this review were carried out and experienced through digital modes of interaction, they cannot necessarily be considered as disembodied or less harmful than traditional forms of aggression and abuse • The majority of the studies included in this review used a quantitative cross-sectional approach, and all included studies were conducted in North America or Europe. To better understand adverse digital experiences and the long-term effects of such experiences for adolescents, longitudinal as well as qualitative research methods should be employed in producing nuanced and in-depth knowledge • Future research on adolescent’s adverse digital experiences within intimate partner relations should to a greater extent explore experiences and consequences in terms of gender to contribute to the existing published research • School nursing staff, teachers, and parents should be offered training which enables them to effectively communicate with and support adolescents in relation to digital safety and ways of interacting and communicating safely within intimate relationships • School curriculums should include age-appropriate content too foster the digital competence of adolescents, particularly with regards to digital safety and knowledge about appropriate and harmful digital conduct within intimate relationships

*Note.* Implications for practice, policy, and research.

## Conclusions

IPV among adolescents has been internationally recognized as a considerable public health concern. In recent years, modes of interacting digitally have provided new ways of carrying out and experiencing abuse in intimate relationships. This scoping review aims to identify the range and nature of adverse experiences and consequences experienced digitally in the context of adolescents’ intimate relationships.

Overall, the most common adverse experience among adolescents identified in the studies included in this review was being controlled by a partner. Furthermore, harassing experiences such as electronic intrusiveness, violations of privacy, receiving an excessive number of texts from a partner, or receiving messages that included insults or content experienced as frightening were common.

The findings of this review are that the current research indicates that many young adolescents and adolescents experience a range of mild to severe adverse experiences, and that such experiences can have negative consequences for their physical, emotional, or mental health. The included studies explore, to a limited extent, the relationship between the adolescent’s adverse experiences and the type of consequences and effects DDV and DDA can have in the short and long term.

However, a significant limitation identified in this scoping review is the need for longitudinal studies assessing the long-term effects of experiencing specific forms of DDA/DDV in childhood or adolescence. As a field of research, this area could benefit from studies that employ longitudinal as well as qualitative research methods, and studies based on first-hand experiences, to produce nuanced and in-depth knowledge about the adverse digital experiences of adolescents within intimate partner relationships, and the kinds of short- and long-term consequences that different types of adverse experiences may have on their health.
